# The Murderer's Confession
*Letter to the Right Hon. William E. Gladstone, M.P., with the Address to the Jury by his Honour Mr. Justice Therry, at the Opening of the First Circuit Court, at Brisbane, Moreton Bay, May 13, 1850; and his Speech at the Dinner given to the Judge and Members of the Circuit, by the Magistracy and Gentry of the District. Sydney: Printed by Kemp and Fairfax. London: J. Ridgway, Piccadilly. 1850.


**Published:** 1851-10-01

**Authors:** 


### ART. VI.—THE MURDERER'S CONFESSION.*

THE subject of crime—in all its revolting, painful, and varied aspects—is a legitimate matter for psychological investigation. It has often occurred to us that much valuable light would be thrown upon the workings of the healthy and morbid mind, if we could obtain ready access to the legitimate and truthful confessions of those who have been engaged in the commission of great crimes. Such documents are at all times difficult to obtain, and when, as it occasionally occurs, a criminal who is on the eve of forfeiting his life on the gallows is induced to make a confession, and to develop in detail, the motives which led to the perpetration of his crime, we are compelled of necessity to look with some suspicion upon his statements, and to admit their truth with considerable limitations. That confessions are sometimes made in which the most implicit confidence may be placed, we have no doubt; and when these are to be procured, they constitute valuable *data* for the study of the jurist and medical psychologist.

We have before us an extraordinary document in relation to this

* Letter to the Right Hon. William E. Gladstone, M.P., with the Address to the Jury by his Honour Mr. Justice Therry, at the Opening of the First Circuit Court, at Brisbane, Moreton Bay, May 13, 1850; and his Speech at the Dinner given to the Judge and Members of the Circuit, by the Magistracy and Gentry of the District. Sydney: Printed by Kemp and Fairfax. London: J. Ridgway, Piccadilly. 1850.

subject. It contains, perhaps, one of the most remarkable criminal confessions upon record. It is cited by the author of the pamphlet, to establish the importance of preventing, by every possible means within the reach of the law, the guilty from escaping certain punishment. In illustration of his views on this subject, the author gives the following particulars:—

"About the year 1835, when I happened to hold the office of Assistant Crown Prosecutor, it was allotted to me to conduct a prosecution against several persons,—servants on an estate near Berrima,—charged with the murder of a man in the same employment. The trial lasted the whole day, and the evidence variously affected the prisoners; but there was one of the prisoners—John Lynch—on whom the evidence had fixed a more prominent part in the perpetration of the deed than the others. Towards the close of the trial, a very material witness, and one who was to have proved that Lynch had been seen, on the day of the murder, within a short distance of the spot on which an attempt was made to consume the body by fire, and on other points to bring guilt completely home to him, appeared in the witness-box in such a state of intemperance that his testimony was valueless. To that incident I attributed it—as I did not hesitate at the time to avow—that the prosecution failed, and Lynch with the others was acquitted. 'Tis true, the presiding judge (the present Sir William Burton) most deservedly imposed a fine of £50 on the delinquent witness, who was the overseer of the estate on which the prisoners were convicts. This occurred in 1835,—and in six years afterwards, during the absence of the present respected Attorney-General in England, his office devolved on me, and it became my duty, at the Berrima Assizes of that year, to prosecute this same Lynch for a murder perpetrated under circumstances of great enormity. For that murder he was tried, convicted, and executed. But the worst respecting him remains yet to be told. In the interval between his acquittal in 1835, and his conviction in 1842, he committed nine distinct murders, making the sum of his terrible guilt to amount to ten murders, to which he confessed previous to his execution!!—and in this admitted catalogue of his crimes, he acknowledged that the murder of his fellow-servant, on the estate near Berrima, was one in which he had a principal share. To add the greater guilt to deeds so horrible, these crimes were perpetrated under circumstances of atrocity to which, from his own narrative, taken down from his lips, the records of crime in this or any other country furnish no parallel,—a memorable and dreadful example of the calamity that may befal a community when a man, charged with a serious crime, of which he is guilty, is tried and acquitted, and let loose again upon society—a far more dangerous pest than before—emboldened by impunity with fresh desperation and augmented hardihood to enter anew upon a career of crime, and calculating upon the difficulty of the proof of guilt, of which his experience of the ordeal through which he has lately passed inspires a natural assurance. Grievous as is the wrong—one I believe rarely inflicted—of unmerited punishment being inflicted on an innocent man,—the wrong is grievous, too—and one perhaps of far more frequent occurrence—of guilty men being permitted to escape with impunity; for we must bear in mind that, besides the mercy due to an individual, there is a mercy at least as imperative involved in the justice due to the public, in securing the safety of their lives and the protection of their property."

When speaking of the *cause of crime*, the author observes,—

"For the last seven years I have filled the offices (with the interval of two years' absence in England) of Attorney-General and Judge of the Supreme Court in Port Philip and in Sydney; and the result of that experience supplies to the question—what is the cause of crime?—the answer, 'that intoxication is the hot-bed from which crime springs.' Directly or indirectly, all crime is traceable to it—the exceptions being so few as to establish the general rule. If a dray is stopped and robbed on the highway, what is the first object of search?—the keg of spirits. If there be no spirits, the plundered property is converted into cash, speedily to be spent in intoxication. If a store in the country is robbed, the first plunder is that of the cask or the bottle that contains some intoxicating liquor. A quarrel, that after a short time, with a little reflection, would be forgotten by sober minds, is renewed and revived with fresh exasperation in the mind at a moment of intoxication, and a thirst created for the most disproportionate and dreadful revenge. At such a moment, too, the jealous mind, without any real ground of jealousy, converts remote suspicion into certain conviction, and so on through the whole range of the human passions. Indirectly, intoxication is the cause of crime by producing poverty; for, in this country, habits of inebriety constitute the main cause of it, as no man in this country capable of work is necessitously poor who does not spend in intemperance those means by which he should support his family. Poverty, again, begets crime; and thus from intoxication, as from a parental source, both derive their existence."

These are important facts, and, proceeding from a man of the author's experience as a judge in a penal settlement, where his means of observation must necessarily have been very extensive, they are entitled to our serious consideration in any investigation we may make into the subject of crime. With the view of first showing how drunkenness alters the whole nature of man, and transforms one who, in his sober moments, was a humane man, into a downright demon; and secondly, to establish that it is a vice from the evil consequences of which no rank or class is exempt, and that wherever it prevails, its victim is always doomed—though he may escape an ignominious fate—to poverty, to degradation, and disgrace, the author gives the subjoined details,—

"The criminal was the parish clerk and sexton of St. John's Church, Campbelltown. He was a person accustomed to the observances of religion,—and bore the character of an inoffensive neighbour. It happened, on the occasion of some trivial quarrel with his wife, he repaired to a public house, and there becoming maddened with liquor, he exclaimed, 'Give me one half-pint of rum more—it is the last I shall ever drink.' Within an hour from having drank that last disastrous draught, he imbued his guilty hands in the blood of his wife and two children as they slept. For this monstrous crime, an ignominious expiation of his life was made upon the scaffold. Yet, when the morning sun arose upon the day on which he did this foul and damning deed, there was as little reason to suppose that ere its close he would have committed an act of such atrocity, as that any who now hear me, will this day be guilty of a like terrible perpetration. This is the history of a drunkard's deed, and a drunkard's doom.

"The second instance to which I refer is one of a painful, though less revolting character. It is the case of a person who was summoned before me,—when Commissioner of the Court of Requests of this colony,—for a debt of £10. The defendant had been a field-officer, and had led into action one of our most gallant regiments in a memorable battle fought during the Peninsular war. He admitted the plaintiff's claim; it was a debt due to a baker for bread supplied to the defendant and his family. On being asked how he proposed to satisfy the demand, he said—drawing from his pocket the gold medal awarded to field officers who led regiments into action at the battle of Albuera—'This is all that is left to me—I have no other means of liquidating the debt.' He then handed the medal to the plaintiff, who paused but for a moment, and with a prompt generosity that I never shall forget, and that touched deeply the feelings of all who heard him, addressed the defendant—'No; you have won that medal nobly in the service of your country, and it shall never be said that I deprived you of it. I forgive you the debt, and, moreover, whenever you want a loaf of bread for yourself or the family, come to me and you shall not go without it.' The whole scene was truly affecting,—and one is at a loss whether more to admire the noble generosity of the plaintiff, or to pity and deplore the degradation and deep sense of self-humiliation endured by the defendant,—a brave soldier, and a gentleman possessed of many accomplishments,—yet who, it was well known, by habits of intemperance had reduced himself to a state of poverty that obliged him to accept of the humble baker's bounty, for the supply of the first necessary of life to his family."

We now purpose laying before our readers the confession of the murderer LYNCH in detail. The guilty career of this man—originally transported from Ireland—is perhaps unequalled in the history of human crime. Any one of the dark deeds he committed would deservedly entitle him to be ranked amongst the Thurtells, Rushes, and other most execrated names in the criminal calendar:—but the *series of such deeds*—which he admits he perpetrated with cold-blooded atrocity—defies the page of history to produce a parallel. At the time of his trial he was, apparently, about thirty-two years of age. His appearance and manner were not only not of a forbidding, but of a mild and prepossessing character. Though undefended by counsel—he conducted his own defence with self-possession and coolness, and with remarkable ingenuity. In his confession, he first minutely detailed the circumstances attendant on the murder of two persons whom he met on the road, and whose company he joined. These two persons—one a black boy—were driving a dray belonging to Mr. T. Cowper, laden with bacon and other articles for the Sydney market. Lynch killed them with an axe as they lay asleep—hid the bodies under a heap of stones—proceeded to Sydney, and sold the articles on the dray in Sydney, and on his return up the country in sole charge of this empty dray—he proceeded to state—he fell in with two Frasers (father and son), and thus describes his manner of making their acquaintance, and his mode of dealing with them:—

"While encamped at Bolland's, at the Stone-quarry, the two Frasers came up with Bawtree's horse team and dray. We sat together by the fire, had a great deal of talk, and, as usual, I told them as many lies as suited my purpose, and managed to get from them an account of the whole of their and their master's concerns, as well as of the valuable load they had on. But I had then no intention of doing them any harm. We travelled together next day, and I was enabled to afford them great assistance in getting on their tired horses, for neither of them could drive well, and I was clever in the management of draft cattle. We encamped in Bargo Brush, by the side of the road, and a cart with two men and a woman afterwards joined us. When we were all lying down, and, I believe, all but myself asleep, a man on horseback rode up, and made particular inquiries about Mr. Cowper's dray, describing it exactly, and telling the whole history of its disappearance, as well as that of the driver and black boy. I lay still, and did not speak a word; but Fraser, the old man, got up, between asleep and awake, and answered something at random. The rider then asked the distance to the nearest inn, and went on. '*Whew!*' said I, 'this is sharp work,—this will never do,—I must get rid of this dray, and obtain another somehow.' I had the whole night to think over the matter, and to form my plans. So in the morning I went, under the pretence of looking for my bullocks, but in reality of driving them away into a deep gully. I strangled the dog belonging to Mr. Cowper's bullock driver, and staid away long enough to allow the other cart with the two men and the woman to leave,—knowing that the Frasers, who seemed greatly to desire my company from the assistance I could give them in managing their teams, would wait for me."

"When I returned, I told them that my bullocks were nowhere to be found, and I had no doubt they had gone to their own home—up the country beyond Berrima. I consulted with them what I had best do, and we agreed that I should leave my dray there, since it was nearly empty, and go on with them for the bullocks, as they offered to take the few things I had on their dray. We encamped for this night in a flat on the other side of Cordeaux's Hill.

"He did not tell me, writes the magistrate, why he allowed the night to pass without perpetrating the intended murder of the Frasers, but—'in the morning,' said he, 'young Fraser went over the ridge to get in the horses, and I volunteered to go with him and assist. It was cold, and I put on a pea-jacket—not to keep me warm, however, but to conceal an axe which I held under my arm. When I got up to young Fraser, I had no difficulty in obtaining the opportunity I wanted. I gave him one crack on the head, and he just dropped like a log of wood. *If people knew how easy it is to take away life, things of this kind would happen oftener.*'"

In cases of suicide, the presence of means for the easy commission of the act has been known often to originate the impulse. The bare sight of blood has given rise, in a particular order of mind, to feelings of extreme mental agony, and has suggested ideas quite opposed to the natural thoughts of the individual. A gentleman, subject to great mental depression, made an attempt, but an unsuccessful one, to cut his throat. He assured us that he had no idea of injuring himself until one day whilst shav'ng he cut his chin, and caught the sight of blood. The impulse then immediately seized him, and he applied the razor, and made a fearful gash in his throat.

There can be no doubt that one of the great means in our power of lessening the amount of serious crime is to render, in every possible way, the act difficult of accomplishment. The facility offered for the purchase of some of the most deadly poisons, prior to the passing of

Lord Campbell's bill relating to the sale of arsenic, led to the commission of many capital offences:—

"I then returned to old Fraser, who remained with the dray, and began yarning to him. After a time he began to wonder what had become of 'Wully.' I had my axe all right, but would not strike until I could make sure. At last he turned his head, and down he went. The next business to attend to was the getting rid of the bodies. I dragged the old one some yards out of the way, lest persons passing through the flat might come upon it, and then returned to the body of the son. With a spade I got from the dray, I dug a hole and buried him; afterwards, I buried the father in the same manner.

"By the time I had finished, the day was far advanced, so I thought it better to stop there for the night. By the evening of the next day I got to Mulligan's. I had no notion of trusting them, or indeed anybody, so I amused them with an account of my being hired to drive up the dray for a gentleman in Sydney. The family consisted of the old man, Mulligan, Mrs. Mulligan, who lived with him but was not his wife, her son, a lad of about eighteen, and her daughter, a girl of about fourteen. Mrs. Mulligan seeing chests of tea on the dray, said she was out of tea, and proposed to buy a chest of me. At first I reminded her of the bad faith they had kept with me before, but pretended at length to bargain with her. But this was only my craft, you should know, for I had no notion of letting her have any of the property: I only wanted to know how much money they had in the house. She said they had £9. We did not come to any agreement, but I let them hope they would get some of my master's property. The next day, in the afternoon, I pulled out a note (£1) and sent to Gray's public-house for rum to treat them. In the evening we drank together and got very sociable, but I took care not to drink much. Well! thoughts were in my head, and the time was coming on; I began to feel very disturbed, and I walked out of the hut. It was a clear, cold, windy night, I looked up at the bright moon, and I prayed to Almighty God to direct me!! I said to myself, I am an injured man, and the Mulligans have defrauded me of what I perilled life and liberty to obtain. That fellow, when I was starving in the Berrima Iron Gang, has often passed me by without so much as giving me a shilling, when he had many pounds which were justly mine in his hands. And now, would it not be right that they should lose all they possess, as a judgment upon them for withholding his own from the poor prisoner? Heaven guide me, and point out to me what to do!* Well, I went into the house again, and we had another glass of rum round. Now it was a cold, windy night, so I took up the axe and said I would go and cut a few barrow-loads of wood for the fire, if John (meaning the young man) would wheel them in. We went out and had some talk whilst I was cutting up. He said that Mulligan was an old man, that he should have the farm at his death, and that God Almighty would soon take him away, adding that if he did not, he (John) would give him (mayhap) a helping hand. I was shocked to hear him speak in this way, knowing how near he was to his own departure out of this world; so I said, 'Ah! John, you should not speak in that way; you don't know what may be in store for yourself.' At this time he had taken in two loads in the barrow, and was come for the third. I had just finished my work, so I took the axe, gave it a back-handed swing against his skull, and threw it down. I threw a quantity of boughs over the body,

* This profane and impious expression contrasts strangely with the temptation, under which he acted, when he murdered Mr. Cowper's drayman and the black boy. He there states—"If there be such a thing as the devil, he was busy with me, and would not leave me alone: it was as if somebody was tugging at me."

and went back to the hut. We had another glass together, and the mother inquired for her son. I said he had offered to go into the bush to see if my horses were right. After a time she began to wonder that John did not come back, and to be very fidgety. This bothered me. She also mentioned a dream she had the night before: she thought she had an infant child, and that she had seen this child horribly mangled and covered with blood. I hated this old woman, for she used to toss cups and balls, and could foretel things. Well, nothing would satisfy her but she must go to the door and cooey.* She *cooeyed* for John, but no John came; and at last she would not even drink, Then old Mulligan said, 'Perhaps the lad is lost in the bush;' and took his gun outside to fire, for the purpose of directing him as to the position of the hut. It did not suit me to have neighbours drawn to the house, so I said to Mulligan, 'You had better not fire; people will come—perhaps the police; and if we are to deal, it wont answer that the dray should be seen here.' 'Truth, lad, that's a right thought of you,' he answered; and instead of firing, folded his arms, holding the gun with the muzzle pointing up. Well, there was no quieting the old woman, and I had my eye upon her inside, at the same time that I was standing by Mulligan outside. *I saw her take out a large knife and conceal it in her own clothes,* and then give it to the little girl. There was no time to be lost."

What were the intentions of the old woman? Had she a suspicion that there had been foul play with her son, and did she seize the knife anticipating a struggle with Lynch for her own life?

"I had left the axe on the ground when I had cut the wood, but my own, with which I had such good luck with the other four, was in the dray; but then, how to get it without showing my intent—but I never was at a loss in the scheming line, so I pretended that a dog I had got was troublesome, and took him to tie him to the wheel of the dray; this gave me an opportunity of getting the axe, and placing it unperceived under my thick coat. By this time the old woman, who seemed bewitched, would be content with nothing short of going outside and looking for her son; she went towards the spot and began moving the boughs which covered the body. Now or never, thought I—I prayed to God to help me!!!—determined to succeed or perish in the attempt—and kept my eye upon Mulligan, who was close beside me: he turned his head—one blow, and down he went. I then hastened towards the old woman—she was in the act of returning, having found her son's body; but, playing the cunning, she said, 'Lord! what brings the police here?—there are three of them getting over the fence.' I was not to be gulled that way, so I gave her my foot, which staggered her, and then brought her down. None now remained but the little girl. The poor little thing had never done me any injury, and I was really sorry for her. I went into the hut where she remained, and I said to her—'Now, my little girl! I will do for you what I would not for the others, for you're a good girl: you shall have ten minutes to say your prayers."

"Here (says the magistrate) Lynch paused, as if he had a difficulty in going on. I suppose it might be a feeling of remorse; and I could easily imagine that the scene of the child begging for life must have been a most pitiable one. I therefore ended the pause by saying—'In short, you killed her, and with the axe.' He said, 'I did;' upon which I bid him proceed. 'I now,' he proceeded, 'began to consult with myself as to the

* A common mode of calling in Australia, which by keeping up a long drawl on the first syllable *coo*—and uttering the last syllable *ey*, in a loud and sharp tone, is heard at a great distance in an Australian forest.

best mode of disposing of the bodies. If I buried them in a frequented neighbourhood like that, it was likely that the graves might be discovered. There were plenty of Wombat* holes near at hand, but it would be troublesome to carry all the bodies, and the native dogs might pull them partly out. I felt an aversion to the thought of burning the bodies of my fellow-creatures—*it seemed such inhumanity*."

One's blood boils at the idea of an impious wretch like this talking of "humanity," just as he was on the eve of burning the bodies of three of his murdered victims!

"But then," he continues, "I considered that the poor things could feel nothing, and that it was little odds to them whether they were burned or buried. I therefore put them upon a heap of logs close to the house, where the Mulligans had been burning off a piece for potatoes. When the fire was well made up, I was surprised to see how the bodies burned. They flared up as if they were so many bags filled with fat. It was an awful thing to stand alone in the dead of night, and to see the four bodies burning to ashes. By morning there was nothing left but a heap, like of slacked lime; I took it up in my hand, and buried it in another part of the paddock. I may have left, perhaps, some ends of bone behind. I then burned the greater part of the Mulligans' clothes, and made such alterations in the house as I judged necessary; for I had still a difficult card to play, and must satisfy the neighbourhood that I had become rightfully possessed of the farm, horses, and cattle.

"The first thing I did was to go to Gray's inn and ask to see him. On his coming out, I inquired of him, with seeming concern, what kind of a man Mulligan was in his dealings. I knew his answer would be an inquiry why I asked. I said that I had just come from Sydney, where I had met Mulligan and concluded a bargain with him, but that he had failed to deliver the cattle as he had promised. Knowing by Mulligan's papers the persons to whom he probably owed money, I took care to go to them, and make similar inquiries. Some of them seemed to look down upon me as a kind of flat, and that Mulligan had taken me in. I acknowledged that I had lent him a valuable mare, which had cost me eighty guineas, and pretended to look very blank when it was hinted that perhaps I might never see my mare again. Some thought that the whole thing was made up between Mulligan and his landlord, 'Smith,' for some fraudulent purpose of their own.

"I then went to Sydney, called at the *Gazette* Office, and pretending to be Mulligan, paid for an advertisement in his name, to the effect that his wife having absconded from her home, he would not be answerable for her debts. I then wrote several letters in his name to persons in the neighbourhood of his farm, being chiefly arrangements about money matters—for I had collected enough of his affairs to be able to word them in a suitable manner. When I returned to Wombat Brush, all these things were told me as so much news, and I appeared to be a victim. I afterwards wrote a letter to myself, in Mulligan's name, as from Illawarra, and employed a man to put it for me in the Campbelltown post-office, and this I showed about. The stupid fellow, however, not knowing my meaning, put it in Liverpool instead, and thus the post-mark," he added, smiling, "enabled you, sir, to detect the trick after I was apprehended. But it answered well with the neighbours. A man on the next farm, who at first troubled me with a great many awkward questions, was at length so satisfied that all was right, that he wanted me to marry his daughter.

* A small quadruped, peculiar to New South Wales, that burrows in the ground, somewhat in the manner of rabbits in England.

"I have mentioned these things all at once, to account for my being allowed to enter, without dispute, into the possession of the Mulligans' property. I succeeded in throwing dust into everybody's eyes. Even the officer of the mounted police, and his three troopers, who called at the hut a few days after the murder, went away quite satisfied. But before I took all these steps I went down to Appin, with a light cart and two of Bawtree's horses, to fetch up Barnett and his wife, who had been fellow-servants with me at M'Evoy's there. I had promised, when I parted with them, if I met a situation they would like, I would let them know. I accordingly described to them the Mulligan family, and hired them in Mulligan's name. I left them the cart and one horse to bring them up. I selected them because they were immigrants, and simple people. They would believe anything you told them. I had therefore no difficulty in accounting to them for the absence of the Mulligan family. I told them that Mulligan and his wife had had a row, and that he had turned them out; and that he had been obliged to go to the Five Islands and hide, on account of a horse found in his possession which was all wrong (that is, stolen)."

It would appear that the murderer was quite destitute of all feeling of remorse, or compunctions of conscience, for after wading through a sea of human blood, he expresses himself as being "*comfortably settled*" in the house of the man whom he had, with his *wife*, *son*, and *daughter*, butchered in cold blood! It is a surprise to us that the stones with which the house was built did not crumble together, and crush this monster.

"*I was now comfortably settled*, made improvements on the farm, determined to clear and fence an additional paddock, and intended to live honestly and do everything fair and square, but I was obliged to go down the country to settle things with Smith, then the landlord. He was a knowing shaver—but I was at least as deep as he—so we arranged matters to the satisfaction of us both.

"Returning home on one occasion from Sydney, on the 18th February, last, I encamped on the north side of Razor-back. In the morning, while on the point of starting, I was met and accosted by a strange man, who seemed very free and open in his conversation, and said that he wanted to get out of the way, and that it might not be known where he was going. 'Why,' I said, 'you do not look like a bushranger.' 'No,' said he, 'I'm an emigrant from Ireland, and have just quarrelled with my wife, and have sworn never to live with her again.' I wanted a man to help to put up some fencing. Now this was a pretty (i. e., strong-built) man, and had the look of a good man for working, and being simple enough to all appearance, seemed just the fellow to suit me. I spoke him fair, and after some more talk I hired him for six months for 15*l.* This was Kerns Landregan, the man on whose account I am about to suffer. At the time I speak, I had not the most distant intention of doing him an injury. We proceeded together towards Berrima. I gathered more from him on the way—he said that he and his wife had earned together as much as 8*l.* per week, up the country, during the last harvest. On my saying it was a large sum, he said that he could work against any man, and his wife was accustomed to work too. She used to bind the sheaves for him. On his parting with her he had stuck to the money (i. e., kept it himself). I said, 'Is she your lawful wife?' He replied, 'Yes.' 'And can you,' said I, 'defraud your own lawful wife of the money she has hardly earned by the sweat of her brow? I would myself take a musket and rob upon the highway sooner than be guilty of such cruelty. I tried to persuade him to give her some of his money, but he was obstinate. When we passed Bolland's, where his wife was staying, I saw her, while he hid himself under some clothes in my cart. I then again tried to persuade him to give his wife her own proper share, since he had parted from her, as he declared, for ever. But he had no feeling for her, and my heart began to turn against him, and to feel a hatred for him as a selfish and hardhearted man. When we got to Crisp's, he hid himself again, and on my asking all about it as we got on the road, he gave me an account of his having accused Mr. Crisp, before the magistrates, of stealing a bundle that he had left at the house. From his account I perceived he was a kind of lawyer, and fond of court. Besides, on getting better acquainted with him, I found he was by no means simple, as I at first supposed, but had a deal of cunning about him. I was sorry that I had hired him, and would have got rid of him at once, but, as ill luck would have it, having nothing about me but orders, which I could not get cashed at Stone-quarry, I had borrowed a one-pound note of him—I tried everywhere in vain to change my orders during the day, but could not—I was even obliged to borrow another one-pound note from him. Towards sundown, two men with bundles joined us on this side of Nattai Bridge, and expressed their intention of camping with us for the night. But this did not suit me, so that I spoke roughly to them, on which they were offended and went on. We encamped (Landregan and I) on the spot well known to you, sir, and then I began to think what I should do; I was greatly agitated, and could not close my eyes, while the other fellow slept like a pig. What was I to do? If I took this fellow with his law to the farm with me, it would certainly be my ruin, for after using his wife as he had done, he would not stop at informing against me; even if I got money in Berrima (which I could do), and paid him his two pounds, telling him at the same time to be off, he would have me up to court for a breach of agreement, and then the magistrate might ask questions. We had been seen together by so many people on the road, that there would be a great risk in killing him; but, everything considered, it seemed the safest and best plan after all. He deserved it for his ill usage of his wife, and he had some money in his pocket, although it was not for his money I killed him. I passed the night thinking over these thoughts, and on the next morning, after putting to the horse, I set my eye upon him. He was a powerful made man, I—small, as you see I am; and he had boasted to me that since he was fifteen or sixteen years old he had never met the man that could throw him. Well, my man, thought I, I fancy I shall be able to settle you, notwithstanding your fine limbs. He had just laid down the tomahawk with which he had been cutting a little wood to make up the fire. I took it up without his perceiving me. 'Now I must mind what I am about, for if I do not hit fair, and he tackles with me, I shall be done.' He sat astride on the long log on which our fire was, smoking his pipe, thinking of nothing. His head was a little turned from me; I gave him one blow and he fell, and then another when he was down, but the first settled him. I then hid the body under some bushes, where it was found next day, stripping off all the clothes to the shirt, and hiding them. I intended to have returned as soon as I conveniently could, and buried the body; but my time was come, and I can see the hand of God in my detection, for I well remember taking off the belt (the discovery of which in my house was the strongest thing against me at the trial) and throwing it into a small hole of water; but afterwards perceiving the end of it above the water, and fearing to leave it there, I pitched it into the cart, and never thought of it since. This was Sunday. I returned home, and on the Tuesday I was apprehended by your orders. You know, sir, how, by degrees, everything then came out."

The preceding melancholy, humiliating, and revolting statement affords data for grave and serious comment. The most charitable construction to put upon the matter would be, to suppose what the criminal himself imagined, that the *devil* had actually got possession of his mind; for he observed, when alluding to the murder of Mr. Cowper's drayman and the black boy, "*if there be such a thing as the devil,* HE WAS BUSY WITH ME, AND WOULD NOT LEAVE ME ALONE: IT WAS AS IF SOMETHING WAS TUGGING AT ME." How fearful to contemplate the impious prayer offered up to an offended DEITY, at the moment when the murderer's hand, still red with human blood, was in the act of being lifted to deprive another fellow-creature of life, sending him, perhaps, unprepared into the presence of the Great Judge. Contemplating one of his revolting murders, he says—"Well! thoughts were in my head, and the time was coming on; I began *to feel very disturbed,* and I walked out of the hut. It was a clear, cold, windy night, and I looked up at the bright moon, and I PRAYED TO ALMIGHTY GOD TO DIRECT ME." Lynch evidently did not pray that God might, in His INFINITE LOVE and MERCY, turn the current of his murderous thoughts, and open his eyes to a just sense of the fearful abyss of crime into which he was about to plunge, both soul and body; but he actually *prayed that God might direct him in the safe commission of the murder,* for thoughts of the injuries which his intended victim had inflicted upon him at a previous period of his life, and feelings of revenge at the same moment, were uppermost in his mind. Lynch again exclaimed, when he appeared to be somewhat in doubt how to effect the murder, and whether Mulligan ought not to perish because he had formerly acted cruelly towards him—"*Heaven guide me, and point out to me what to do!*"

Can a more affecting picture be conceived than that sketched by the murderer, when he so graphically, and no doubt truthfully, describes the yearnings of Mulligan's heart-broken wife after her poor lost child? When the boy was missing, his mother, Lynch says, "*grew fidgetty,*" and "*bothered me.*" She then gave expression to her surprise that John did not return home; and after describing to Lynch a prophetic dream, about an infant child she had seen horribly mangled and covered with blood, which had disturbed her night's rest, he says of the poor woman—"*nothing would satisfy her, but she must go to the door and cooey.*" After resolving to murder her, and arming himself with the weapon which he had wielded so successfully on former occasions, and with which the foul deed was to be perpetrated, he exclaimed, "*I prayed to God to help me.*" But we have said enough to establish that this man must have been either a maniac, in the ordinary acceptation of the term, or one of those awful instances of terrible brutality, accompanied, as it occasionally is, with a thirst for human blood, with which the world's history unhappily abounds, and in which all MORAL SENSE, *affection*, and *feeling*, appear either to have been destroyed, or never to have existed—in fact, cases of *moral anæsthesia.*

The ordinary channels of communication often bring before us similar illustrations of the melancholy condition of the human intellect. The following remarkable case appeared in the *Times.** The facts appear to be taken from a French paper:—A woman, aged forty, named Anne Valby, was tried four days ago by the Court of Assizes of the Côte-d'Or for the murder of her husband. In early life her conduct was most scandalous, and she had two illegitimate children. She was suspected of having caused the death of one of them by plunging it, when quite young, in cold water. She was also suspected of having occasioned the death of her sister. In 1842 she married an old man named Faiveley, of Comblanchien, and he died six weeks after with violent pains in the bowels. Before his death, she had criminal relations with more than one person. A little after, she married another old man, named Guillaume, of the same place, and, though she had two children by him, her conduct continued to be scandalously licentious. She was accustomed to bring in beggars from the roadside to drink and indulge in orgies with her. She had frequent quarrels with her husband, and used violent threats against him. In the night of the 28th of March last, the neighbours heard them quarrelling and fighting, but paid no attention. Two days after, the woman announced that her "old man" had disappeared, and that she did not know what had become of him. She, however, made no search after him, and after awhile went to an adjacent village to pay a visit. During her absence the body was discovered in the well of her house. She returned just as the neighbours had, with some difficulty, succeeded in getting it up. She passed by it with unconcern, and, while it was being examined, coolly sat down and partook of food. Traces of blood were found on the bed, on the walls, and on an axe, and the woman was arrested. Her children, a boy of thirteen and a girl of seven, then stated that they had been awakened on the night of the 28th of March by the quarrel and fight of their father and mother; that the latter had beaten the old man on the head with an axe until he fell on the bed and died; that she had then made the boy assist her in dragging the body to a dark corner, and in placing some furniture before it to prevent it from being seen; that she had afterwards washed the blood out of the

* September, 1851

sheets, and made him and the girl sleep in the bed from which the corpse had been removed; that the next morning she had kept them at home until all the neighbours had gone into the fields, and had then made them assist her in tossing the body into the well. The principal witness against the woman was her own son; but, though his evidence caused a thrill of horror through the court, it produced no effect on her. The jury declared her *guilty,* without extenuating circumstances, and the court condemned her to death. *She displayed no emotion on hearing the sentence, and walked away with a firm step. The next day she was heard singing and dancing in her cell.* How humiliating to the proud reason of man—to his "Godlike intellect"—that he should ever, in obedience to the inscrutable decrees of Providence, be reduced to so sad and terrible a condition! The subject is suggestive of solemn reflections.

